# Conversion of Anergic T Cells Into Foxp3^-^ IL-10^+^ Regulatory T Cells by a Second Antigen Stimulus *In Vivo*

**DOI:** 10.3389/fimmu.2021.704578

**Published:** 2021-06-25

**Authors:** Anna Sophie Thomann, Theresa Schneider, Laura Cyran, Ina Nathalie Eckert, Andreas Kerstan, Manfred B. Lutz

**Affiliations:** ^1^ Institute for Virology and Immunobiology, University of Würzburg, Würzburg, Germany; ^2^ Department of Dermatology, Venereology and Allergology, University Hospital Würzburg, Würzburg, Germany

**Keywords:** T cells, anergy, Tr1, conversion, *in vivo*

## Abstract

T cell anergy is a common mechanism of T cell tolerance. However, although anergic T cells are retained for longer time periods in their hosts, they remain functionally passive. Here, we describe the induction of anergic CD4^+^ T cells *in vivo* by intravenous application of high doses of antigen and their subsequent conversion into suppressive Foxp3^-^ IL-10^+^ Tr1 cells but not Foxp3^+^ Tregs. We describe the kinetics of up-regulation of several memory-, anergy- and suppression-related markers such as CD44, CD73, FR4, CD25, CD28, PD-1, Egr-2, Foxp3 and CTLA-4 in this process. The conversion into suppressive Tr1 cells correlates with the transient intracellular CTLA-4 expression and required the restimulation of anergic cells in a short-term time window. Restimulation after longer time periods, when CTLA-4 is down-regulated again retains the anergic state but does not lead to the induction of suppressor function. Our data require further functional investigations but at this stage may suggest a role for anergic T cells as a circulating pool of passive cells that may be re-activated into Tr1 cells upon short-term restimulation with high and systemic doses of antigen. It is tentative to speculate that such a scenario may represent cases of allergen responses in non-allergic individuals.

## Introduction

T cell tolerance mechanisms include the induction of T cell anergy, T cell deletion and regulatory T cell (Treg) functions. Although the molecular details, how anergy is induced and maintained, are increasingly understood (1, 2), anergy is perceived as a passive state with no function. A functional or active role for anergic T cells in tolerance has not been defined. The usefulness to maintain anergic T cells over long periods *in vivo* is unclear.

Anergy was discovered with CD4^+^ Th1 T cell clones that were stimulated with only *via* CD3 antibodies (signal 1 only) without co-stimulation and defined as functional unresponsiveness to further stimulation despite intact antigen presentation by MHC/peptide (signal 1) and full costimulation *via* CD80/CD86 (signal 2) (3, 4). Most experimental settings to induce T cell anergy *in vitro*, reported defective IL-2 production and impaired proliferation upon TCR restimulation with or without CD28 costimulation (4–6). This so-called *in vitro* clonal T cell anergy could be reverted to proliferation by addition of high doses of IL-2 since anergic T cell clones highly express CD25 receptors (4). In contrast, IL-2 injection could not restore T cell proliferation *in vivo* (4). Mitogens that circumvent TCR-signalling such as combinations of phorbol 12-myristate 13-acetate (PMA) together with a calcium ionophore ionomycin have also been documented to revert T cell anergy (4).

In all cases, anergy, is a passive state that does not fulfill any active tolerogenic role. Therefore, it remains elusive why anergic T cells are maintained for weeks without obvious further function. Maintaining such T cells with unwanted antigen specificity may represent a pool of cells that harbours the potential risk for conversion into autoimmunity-mediating effectors after re-activation (7, 8). There is experimental evidence that anergic T cells can produce immuno-suppressive IL-10 (9–11). However, the physiological stimuli or cell types and mechanistic details that lead to T cell anergy *in vivo* are not well understood. Tolerogenic dendritic cells (DC) producing IL-10 may induce regulatory function in anergic T cells. *In vitro* exposure or injection of immature or semi-mature DC maturation stages of different DC subsets have been shown to induce either T cell anergy or the development into different subsets of Tregs, such as induced Foxp3^+^ Tregs (iTregs) or Foxp3^-^ IL-10^+^ type 1 regulatory (Tr1) cells (12, 13).

In mice, a single intravenous injection of soluble peptide, superantigen or a neo-self-antigen expression induced T cell anergy (14–18). Subsequent IL-10 production by these anergic T cells has been observed after several intravenous injections of peptides that were captured and presented most likely by immature DCs (19). Anergic CD4^+^ T cells also appear in normal healthy mice. They can be identified by the surface marker profile CD44^high^ CD73^high^ folate receptor 4 (FR4)^high^ and could convert into Foxp3^+^ Tregs after adoptive transfer where they prevented autoimmunity (20, 21).

Previously, we addressed the possibility for a conversion of anergic T cells into Foxp3^-^ Tr1 cells *in vitro*. Here, our *in vivo* data confirm now the *in vitro* observations of the transient induction of CTLA-4 expression after high dose i.v. OVA injection. However, only after two short-term interval injections, the cells acquire a Foxp3^−^ IL-10^+^ Tr1 phenotype and regulatory function. Together, these data suggest that anergic T cells can represent a precursor for Tr1 cells.

## Materials And Methods

### Mice

C57BL/6, B6.OT-II.Rag1^-/-^, BALB/c and DO11.10 mice were bred in the animal facilities of the Institute of Virology and Immunobiology at the University of Würzburg under specific pathogen-free conditions or purchased from Charles River. The IL-10-β-Lactamase reporter mouse strain ITIB (22) was kindly provided by Dr. Ulrike Protzer (Technical University Munich). ITIB mice were crossed with B6.OT-II.Rag1^-/-^ mice and the resulting F1 generation was further crossed to obtain ITIB.OT-II and ITIB.OT-II.Rag1^-/-^ mice. Animal experiments were performed after approval and under control of the local authorities (Regierung von Unterfranken, AZ 52/14).

### CCF4-Substrate Loading and Antibody Staining

For CCF4-substrate loading, 1x10^6^ cells were resuspended in R10 medium (RPMI 1640 (Sigma) with 10% heat-inactivated FCS (Gibco), 100 µg/ml Penicillin-Streptomycin, 2 mM L-glutamine and 50 µM 2-mercaptoethanol (all Sigma)) containing 1.3 µM CCF solution (Invitrogen) and 3.6 µM Probenecid (Sigma) and incubated for 90 min at RT in the dark. After incubation, the cells were washed with FACS buffer and subsequently stained in FACS buffer containing 10% 2.4G2 hybridoma cell line supernatant (anti-Fcγ-RII/III, as Fc block). Surface staining was performed with antibodies to CD25 (PC61), CD28 (E18), CD4 (GK1.5), CD44 (IM7), CD73 (TY/11.8), FR4 (12A5), PD-1 (RMP1-30), Vα2 (B20.1), DO11.10 TCR clonotype (KJ1-26) (all BioLegend) and Vβ5.1, 5.2 (MR9-4) (BD Biosciences). In the case of staining with biotinylated antibodies, surface staining was followed by a second incubation with fluorophore-conjugated streptavidin. Subsequently, the cells were fixed with 1% formaldehyde and analysed on a BD LSR II flow cytometer.

### Intracellular and Intranuclear Staining

Cells were stimulated in R10 medium containing 10 ng/ml PMA, 1 µg/ml Ionomycin and 5 µg/ml Brefeldin A (all Sigma) for 4 hours at 37°C. After CCF4-substrate loading and surface staining, the cells were fixed and permeabilized using the Foxp3/Transcription Factor Fixation/Permeabilization Concentrate and Diluent (eBioscience) according to manufacturer’s instructions. Intracellular and intranuclear staining was performed in 1x Permeabilization Buffer (eBioscience) using the following antibodies: CTLA-4 (UC10-4B9), Foxp3 (150D), IFN-γ (XMG1.2), Ki-67 (16A8) (all BioLegend) and Egr-2 (erongr2, eBioscience).

### Adoptive Transfers and Immunizations

Adoptive T cell transfer was performed by i.v. injection of 1-2x10^7^ bulk lymph node and spleen cells from DO11.10 or (ITIB.)OT-II mice or 4.5-7x10^6^ (ITIB.)OT-II.Rag1^-/-^ cells into BALB/c or C57BL/6 recipient mice, respectively. One day later, anergy was induced by injection of 275 µg OVA_327-399_ peptide (China Peptides) or 400-1000 µg OVA protein (Profos, Endograde, endotoxin-free). Control mice received PBS injections instead of OVA. For Tr1 conversion experiments, the mice received a second OVA injections 3 days (short interval) or 11 days (long interval) after the first one.

### *Ex Vivo* Proliferation and Suppression Assays

TCR transgenic DO11.10 T cell transfers were performed as indicated. One day later animals were injected once or a second time with OVA using the same dose and route after 3 days (short interval) or 11 days (long interval) or PBS. For anergy determination, 3 days after the last OVA injection erythrocyte-lysed spleen cells were labelled with CFSE and restimulated with 100 U/ml mouse IL-2 (Peprotech), anti-CD3 alone or anti-CD3 and anti-CD28 antibodies (2.5 µg/ml each, soluble, LEAF quality, BioLegend) or 10 µM OVA peptide. FACS analysis for CD4, KJ1-26 and CFSE of spleen cells was performed after 5 days to analyse the proliferation history of the DO11.10 T cells. For determination of suppression *in vivo*, 5 days after the last OVA injection, erythrocyte-lysed spleen cells were directly FACS analysed for their frequency of CD4^+^ KJ1-26^+^ and further for the intracellular CTLA-4 or intranuclear Foxp3 among them. For determination of suppression *in vitro*, 5 days after the last OVA injection, erythrocyte-lysed spleen cells were enriched for CD4^+^ cells by magnetic cell separation (MACS) according to the manufacturer’s instructions. To test their suppressor capacity these CD4^+^ cells were added to CFSE-labelled MACS-enriched CD4^+^ T cells from BALB/c lymph nodes stimulated with anti-CD3 antibodies and irradiated spleen APCs as we performed before (23). After 5 days proliferation was detected as CFSE dilution measured by FACS.

### Statistical Analysis

Data are presented as mean ± SEM or SD as indicated in the figure legend. Unpaired t-tests were used for comparison of cytokine production after first and second OVA injection. The statistical differences between more than two groups were calculated using one-way ANOVA with Tukey’s test for multiple comparisons. All statistical analyses were performed using PRISM 7 (GraphPad).

## Results

### Induction of T Cell Anergy Markers on Naïve CD4^+^ OT-II Cells After OVA Peptide or Protein Injection

**Figure 1:**
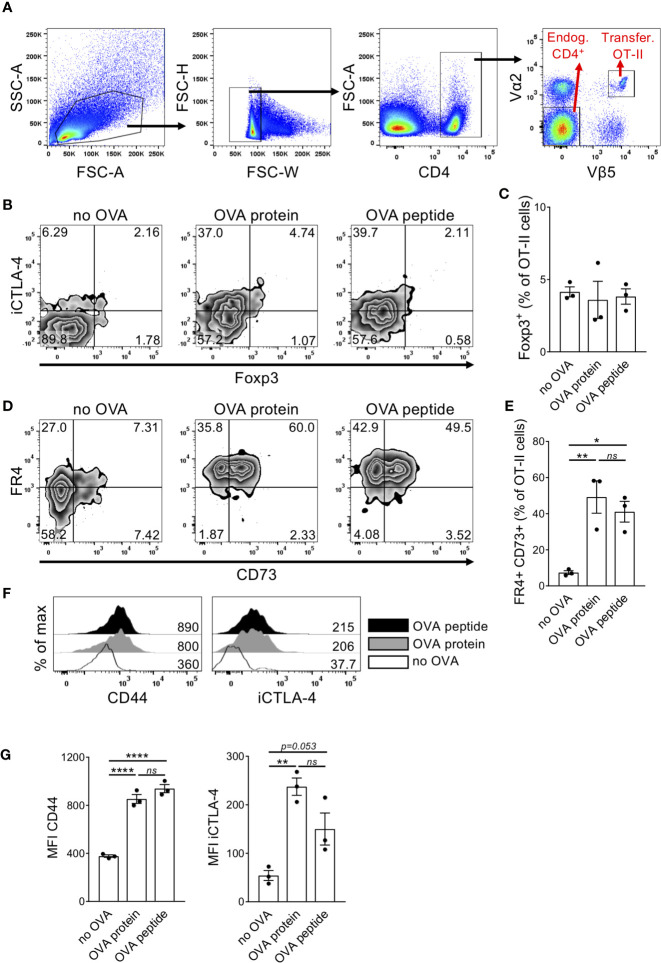
Adoptively transferred naïve T cells upregulate different anergy markers after high dose OVA injection. Transferred OT-II cells isolated from the spleen of recipient mice were tested for expression of different anergy markers 5 days after i.v. OVA_327-339_ peptide or OVA protein injection. **(A)** Gating strategy. Cells were first gated on living singlets. Within the CD4^+^ population, injected OT-II cells can be identified by co-expression of TCR Vα2 and Vβ5 chains, while Vα2^-^ Vβ5^-^ CD4^+^ T cells were chosen as endogenous control cells. **(B)** *Representative FACS plots show expression of intracellular CTLA-4 (iCTLA-4) and Foxp3 in transferred OT-*II cells. **(C)** Quantification of the frequency of Foxp3+ cells in transferred OT-II cells. **(D)** Representative FACS plots show co-expression of FR4 and CD73.**(E)** Quantification of the frequency of FR4^+^CD73^+^ double-positive cells in transferred OT-II cells as mean ± SEM. **(F)** Representative histogram overlays show marker expression in transferred OT-II cells from non-injected (white), OVA_327-339_ protein-injected (grey) or OVA peptide-injected (black) mice. Numbers indicate median fluorescence intensities (MFIs). **(G)** Expression of anergy markers is shown as MFI within OT-II cells summarized as mean ± SEM. Dots represent individual mice. *p < 0.05, **p < 0.01, ****p < 0.0001, one-way ANOVA with Tukey post-test. ns, not significant.

Anergic T cells can be induced *in vivo* by intravenous injection of high dose antigen (14–16) but a characteristic phenotype of anergic T cells is still controversially discussed due to the lack of specific surface markers. A subset of anergic T cells present in steady state healthy mice express CD44, CD73 and FR4 (20). Furthermore, CTLA-4 have been shown to be required for anergy induction and maintenance (24). Intracellular CTLA-4 (iCTLA-4) detection better reflects its kinetics of total expression (25) since surface levels at a given time point are usually very low due to permanent recycling of the molecule (26, 27). To demonstrate anergy induction we used the TCR transgenic OT-II system. After adoptive transfer, injected OT-II cells can be identified by the co-expression of the TCR chain variants Vα2 and Vβ5 (**Figure 1A**). We investigated the expression levels of different anergy markers in adoptively transferred T cells upon i.v. injection of high dose antigen in form of either OVA peptide or OVA protein. Transferred OT-II cells contained less than 5% Foxp3^+^ cells, which did not increase upon OVA injection (**Figures 1B, C**). However, 5 days after OVA injection, OT-II cells co-expressed the surface markers FR4 and CD73 (**Figures 1D, E**). Furthermore, the cells expressed high levels of CD44 and iCTLA-4 (**Figures 1F, G**). Comparing the injection of OVA protein versus peptide, there was no difference in the expression of these markers between the two types of antigen. This indicates that following a protocol for anergy induction after injection of high dose antigen in form of both OVA protein and OVA peptide results in the upregulation of typical anergy markers in adoptively transferred OT-II cells. Since early events of the physiological immune response require antigen processing and presentation, we decided to use the whole OVA protein in subsequent experiments.

### Kinetics of Anergy Marker Up-Regulation

**Figure 2:**
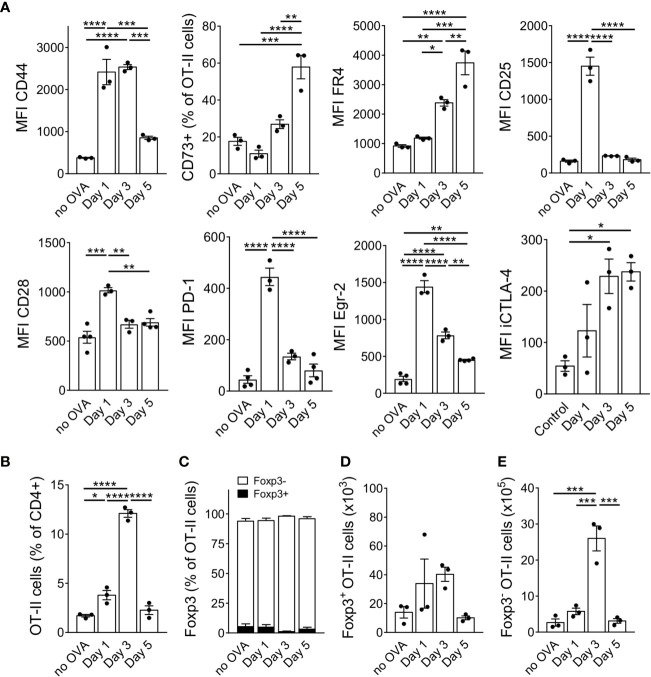
Anergy markers show different kinetics during anergy induction. **(A)** Expression of anergy markers was analysed in adoptively transferred OT-II cells at different time points after a single OVA injection and is shown as MFI when all cells up-regulated the marker in a uniform manner or as frequency when the population split into positive and negative cells for the marker staining (CD73). **(B)** Frequency of OT-II cells at indicated time points. **(C)** Frequency of Foxp3^+^ and Foxp3^-^ cells within the OT-II population at indicated time points. **(D)** Absolute numbers of Foxp3^+^ and **(E)** of Foxp3^-^ cells at indicated time points after OVA protein injections. Data is shown as mean ± SEM. Dots represent individual mice. *p < 0.05, **p < 0.01, ***p < 0.001, ****p < 0.0001, one-way ANOVA with Tukey post-test.

In addition to CD44, FR4 and CD73, others have suggested a combination of antibodies against CD7, CD28 and PD-1 to distinguish anergic T cells from both naïve and activated T cells (28). Furthermore, the transcription factor Egr-2 has been shown to be required for complete anergy induction (29). We therefore analysed these markers at different time points after OVA injection. 24 hours after OVA protein injection, the transferred OT-II cells upregulated CD25, but later downregulated it to levels of unstimulated cells by day 3 after antigen injection (**Figure 2A**). CD44, CD28 and PD-1 were upregulated soon after OVA injection but decreased again by day 5 (CD44) or day 3 (CD28, PD-1). However, their expression remained always slightly above the levels of unstimulated cells (**Figure 2A**). In contrast, CD73 and FR4 continuously increased over time. Surprisingly, the anergy-specific transcription factor Egr-2 also peaked already 24 hours after OVA injection and remained significantly upregulated over the level of control cells. iCTLA-4 was strongly upregulated upon OVA injection and remained at high levels until day 5 similar to the kinetics described *in vitro* (23). After activation and CD25 upregulation, the cells accumulated in the spleen since we observed the highest frequency of OT-II cells (about 12% of CD4^+^ T cells) on day 3 after OVA injection. However, after accumulation, the cells collapsed rapidly again resulting in a frequency of only 2% of OT-II cells within all CD4^+^ T cells on day 5 (**Figure 2B**). As expected, the original ratio of about 95% Foxp3^-^ and 5% Foxp3^+^ cells among the transferred OT-II bulk population remained stable and did not favour Foxp3^+^ cell expansion (**Figure 2C**). The absolute number of Foxp3^+^ cells remained very low and did not increase significantly at any time point after OVA injection (**Figure 2D**). In contrast, the absolute number of Foxp3^-^ cells increased massively at day 3 (**Figure 2E**). This indicates that almost exclusively the Foxp3^-^ population responds to high dose antigen encounter with a massive expansion (**Figure 2E**). Taken together, adoptively transferred OT-II cells were activated and accumulated in the spleen – but rapidly collapsed as well – after high dose OVA injection associated with the upregulation of anergy-associated surface markers and transcription factors acquiring a Foxp3^−^ CD44^+^ CD73^+^ FR4^+^ CTLA-4^+^ Egr-2^+^ CD25^−^ phenotype.

### Maintenance of Anergic Phenotype After a Second Antigen Injection

**Figure 3:**
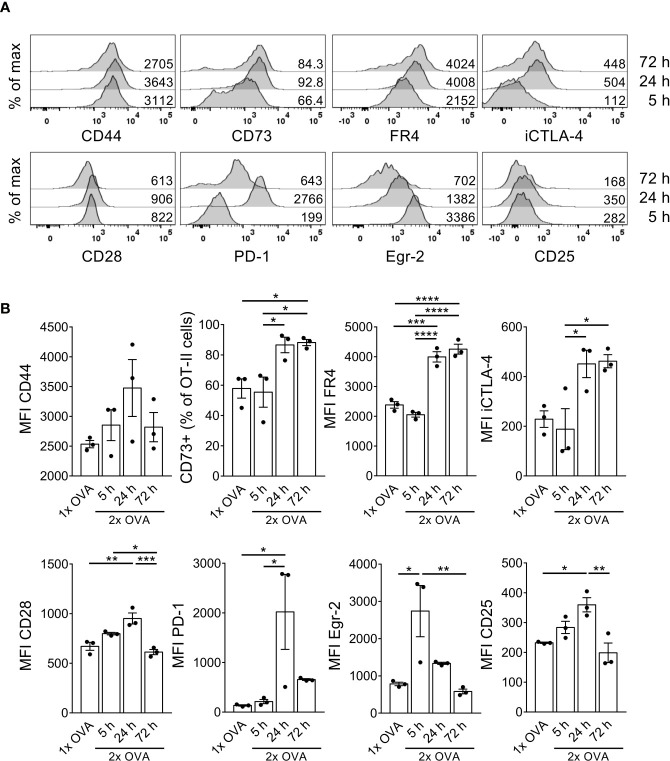
T cells retain their anergic phenotype after a second OVA injection within a short time interval. OT-II.Rag1^-/-^ cells were adoptively transferred into C57BL/6 WT recipient mice and received a first i.v. OVA protein injection 1 day after adoptive transfer. A second OVA injection was given 3 days after the first one. 5, 24 or 72 hours after the second injection, spleen cells were isolated and stained for indicated surface markers. **(A)** Representative histograms depict marker expression in OT-II cells 5 hours (bottom), 24 hours (middle) or 72 hours (top) after the second OVA injection. Numbers indicate MFIs or frequency of CD73^+^ cells within OT-II cells. **(B)** Data from A summarized as mean ± SEM. Dots represent individual mice. *p < 0.05, **p < 0.01, ***p < 0.001, ****p < 0.0001, one-way ANOVA with Tukey post-test.

Stimulation of anergic T cells by immature DCs *in vitro* as well as repetitive administration of antigen *in vivo* has been shown to convert anergic T cells into IL-10-producing Tr1 cells (19, 23). We hypothesized that a second antigenic stimulus must occur within a short time window after the first injection, in which CTLA-4 is highly expressed on the anergic T cells, to allow for conversion into Tr1 cells. Therefore, the mice received a second intravenous OVA injection 3 days after the first one, when the cells had already downregulated CD25 in response to the first injection but expressed high levels of iCTLA-4 (**Figure 2A**). To investigate whether the cells preferentially convert into Foxp3^−^ Tr1 cells or rather Foxp3^+^ Tregs, we now transferred OT-II-Rag1^-/-^ cells, which lack endogenous Foxp3^+^ Tregs. Five hours after the second injection, the cells still showed a phenotype similar to those that had received only one antigen stimulus (**Figures 3A, B**). However, at later time points such as 24 hours or 72 hours after the second injection, the cells further upregulated the anergy markers CD73 and FR4 (**Figures 3A, B**). Other markers such as CD44, CD28, PD-1 or Egr-2 followed kinetics similar to the one observed after only one injection (**Figures 3A, B**) suggesting the maintenance of the anergic phenotype. Of note, the cells displayed a complete unresponsiveness to upregulate the cell activation marker CD25 at any time point upon the second antigen stimulus further strengthening the anergic state.

### Induction of Foxp3^-^ IL-10^+^ Tr1-Like Cells After a Second Injection of Antigen

**Figure 4:**
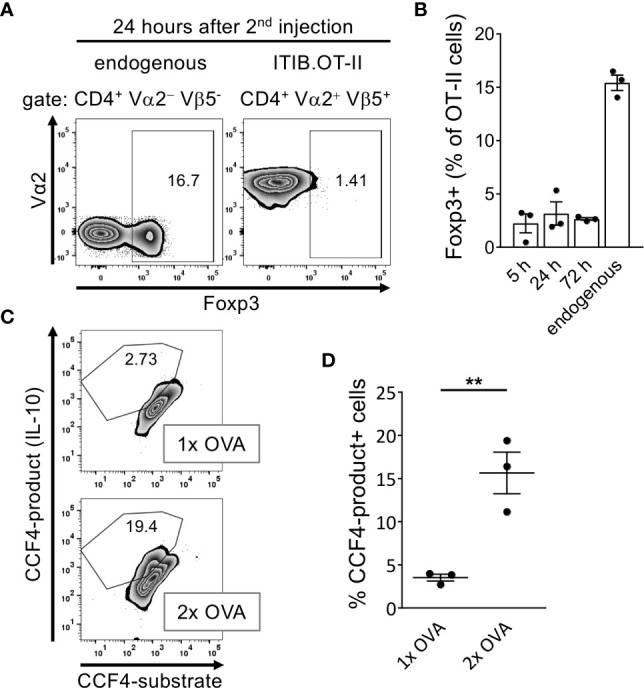
Anergic T cells acquire a Foxp3− IL-10+ Tr1-like phenotype after the second OVA injection. Splenocytes were isolated at indicated time points after the first or second OVA protein injection. **(A)** Representative flow cytometry plots at 24 hours after the second OVA injection show the frequency of Foxp3^+^ cells within the endogenous CD4^+^ or the transferred ITIB.OT-II.Rag1^-/-^ population. **(B)** Frequency of Foxp3^+^ cells within the ITIB.OT-II population at different time points. Dots represent individual mice. The frequencies of Foxp3^+^ cells within the endogenous CD4^+^ populations were obtained at 24 hours after injection but are representative for all time points. **(C)** Splenocytes were tested for IL-10 production by β-Lactamase reporter activity after restimulation with PMA/Ionomycin for 4 hours. Representative flow cytometry plots show the frequency of CCF4^+^ cells within the ITIB.OT-II population 24 hours after the first (upper plot) or second OVA injection (lower plot). **(D)** Frequency of CCF4-product^+^ cells within the ITIB.OT-II population after the first or second OVA injection. Dots represent 3 mice per group. Dots represent individual mice. All data is shown as mean ± SEM, **p < 0.01, unpaired t-test.

Tr1 cells are mainly characterized by the secretion of high levels of IL-10 in the absence of Foxp3 expression (30). We therefore tested our anergic cells for the expression of these markers after the second OVA injection within short time intervals ranging from 5h to 72 h. To measure IL-10 expression, we used T cells derived from OT-II.Rag1^-/-^ mice crossed with an IL-10-β-Lactamase reporter mouse strain (ITIB) (22). We did not observe a substantial Foxp3^+^ population in the adoptively transferred ITIB.OT-II cells at any time point indicating that anergic T cells did not convert into Foxp3^+^ iTregs in our setting (**Figures 4A, B**). IL-10 expression was measured using the β-Lactamase reporter system of the transferred ITIB.OT-II cells. Reporter activity of this enzyme can be detected by using the fluorogenic substrate coumarin-cephalosporin-fluorescein (4)-acetoxymethyl (CCF4-AM) (31). After entering the cell, CCF4 emits green light (520 nm) due to fluorescence resonance energy transfer (FRET) from the coumarin donor to the fluorescein acceptor upon excitation at 409 nm. Enzymatic cleavage of CCF4 by β-Lactamase, however, interrupts this energy transfer leading to blue emission (447 nm) instead (31). Therefore, IL-10 expression directly correlates with the frequency of CCF4-product^+^ cells. 24 h after the second OVA injection the frequency of IL-10^+^ cells almost reached 20% as compared to less than 5% IL-10^+^ ITIB.OT-II cells after a single OVA injection (**Figures 4C, D**).

Taken together, anergic T cells acquire a Foxp3^−^ IL-10^+^ phenotype supporting our hypothesis of conversion into Tr1 cells after a second antigen encounter within a short time interval.

### Transient iCTLA-4 Expression and T Cell Anergy Induction After One or Two OVA Injections

CTLA-4 acts as a negative regulator of T cell responses and is up-regulated in both effector and regulatory T cell subtypes (32) as well as during anergy induction (23, 24). We found before *in vitro* that anergic cells up-regulated CTLA-4 and its high expression was maintained and required for subsequent Tr1 generation. When the second antigen stimulation occurred during the phase when CTLA-4 was already down-regulated again, the cells remained anergic but failed to become IL-10^+^ Tr1 cells (23).

Thus we investigated the question also *in vivo* whether high dose OVA injections would functionally lead to Tr1 conversion only in a short time frame between the two OVA injections when CTLA-4 is still highly expressed, as opposed to a long-term interval when CTLA-4 is down-regulated. Although we used the OT-II system successfully to study T cell anergy to Tr1 conversion *in* vitro (23), the OT-II system did not allow us to follow adoptively transferred and anergized CD4^+^ T cells for longer time periods *in vivo*. Transferred OT-II cells became partially activated showing the expression of anergy markers and expanded after a single OVA injection but finally disappeared thereafter within 7-10 days (not shown). The mild stimulation by OVA injection in the absence of adjuvants did not allow their survival. Anergic cells undergo deletion during the contraction phase, an accompanying mechanism that has been reported to be associated with T cell anergy induction *in vivo* (33). In addition, the OT-II system may have other specific deficits. OT-II cells can be activated and polarized as shown by many groups including ourselves (34). However, they did not differentiate into stable memory T cells, which was in some cases due to peripheral deletion by responses of Vβ5^+^ T cells in C57BL/6 mice to endogenous superantigen derived from Mtv-9 (35), thus including OT-II cells (36). Here, we found that OT-II cells were unable to develop into a stable anergic phenotype after OVA injection and, after a second injection using a long-term interval, most OT-II cells were deleted.

Since the deletion of OT-II cells during the contraction phase precluded further analyses after the second OVA injection. We observed before in another OVA-specific CD4^+^ TCR-transgenic system, that adoptively transferred DO11.10 T cells also contracted after the expansion phase but remained at a stable frequency at least until day 8 after transfer (37). Therefore, and since anergy is a general phenomenon that is not restricted to certain antigens or a specific system of transgenic T cells, we switched to the DO11.10 system.

**Figure 5:**
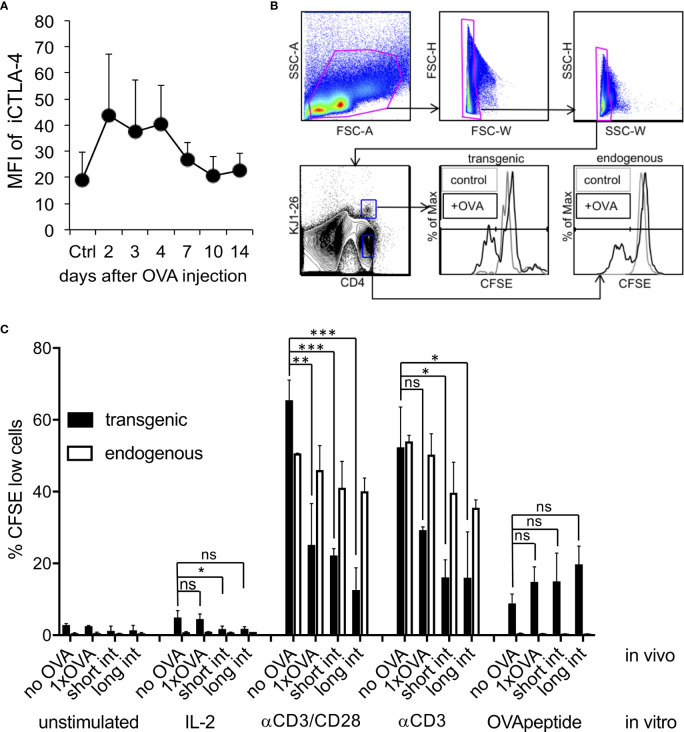
Induction of intracellular CTLA-4 and CD4+ T cell anergy in vivo. DO.11.10 T cells (1x10^7^) injected i.v. into BALB/c mice and one day later with a high dose endotoxin-free OVA protein. **(A)**. FACS analysis of CD4^+^, KJ1-26^+^, Foxp3^-^ cells for their iCTLA-4 mean fluorescence intensity (MFI) of spleen cells was performed at the indicated time points. Control mice received DO11.10 cells but remained without OVA injection (Ctrl). MFI = mean fluorescence intensity (n=3 mice, Error bars: SD). **(B)**. Gating strategy for results shown in **(C)**. Some animals were injected a second time with OVA using the same dose and route after 3 days (short interval) or 11 days (long interval). Seven days after the last OVA injection spleen cells were labelled with CFSE and restimulated ex vivo as indicated. FACS analysis for CD4, KJ1-26 and CFSE of spleen cells was performed to analyse the proliferation history of the DO11.10 T cells. Control mice remained without OVA injection (no OVA). Error bars: SD. Statistics only for transgenic T cells with one-way ANOVA with Tukey’s test for multiple comparisons. *p < 0.05, **p < 0.01, ****p < 0.0001. ns, not significant.

Our previous *in vitro* analysis indicated that generation of anergic and suppressive Tr1 cells only during short-term restimulation critically depended on high CTLA-4 expression, while longer intervals failed to do so due to CTLA-4 down-regulation (23). When we tested iCTLA-4 expression after high dose OVA injection, we found, similar to our *in vitro* data and the OT-II system above, a fast but transient up-regulation of iCTLA-4 in adoptively transferred DO11.10 T cells on day 2 to 4 quickly dropping thereafter (**Figure 5A**).

In addition to upregulation of anergy-related surface markers, *in vivo* induced anergy is characterized by a defect antigen-specific response to restimulation with OVA or using antibodies directed against CD3 or both CD3/CD28 molecules. Other than *in vitro*, *in vivo* anergized T cells do not respond with proliferation on high doses of IL-2 (4). Our data show that a single injection of high dose OVA protein results in functional anergy induction in adoptively transferred DO11.10 T cells in the spleen. We found, that after a single OVA injection DO11.10 transgenic T cells did not respond with proliferation to IL-2, poorly to OVA peptide and much lower than endogenous non-anergized T cells to stimulation with CD3 and CD3/CD28 antibodies (**Figures 5B, C**). To further test whether a second OVA injection would promote the maintenance of the anergic phenotype, we used a short (3 days) or a long-term interval (11 days) for OVA application. Both protocols revealed a sustained anergic state as observed after a single injection of OVA (**Figures 5B, C**). Together, these data indicate that high iCTLA-4 expression correlates with the capacity of anergic T cells to convert into Tr1 cells, whereas using the long-term interval the anergic T cells simply remain anergic.

### Short-Term but Not Long-Term Interval OVA Injections Favour Induction of CD4^+^ iCTLA-4^+^ Foxp3^-^ T Cells With Suppressor Function

**Figure 6:**
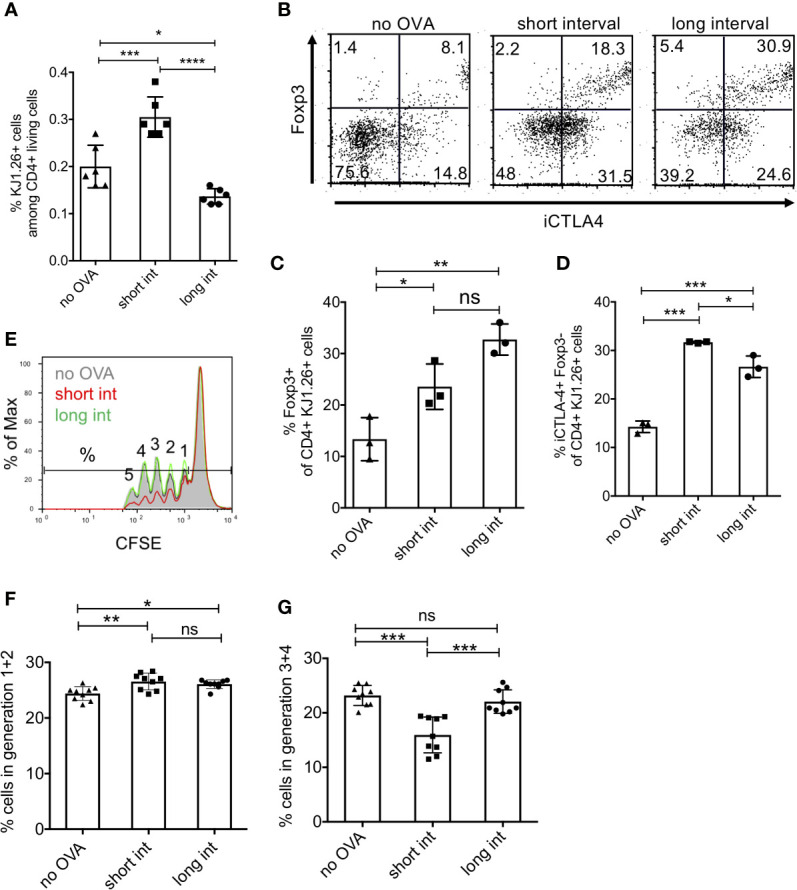
Induction of regulatory T cells is favoured by short interval OVA injection in vivo. DO.11.10 T cells (1x10^7^) were injected i.v. into BALB/c mice and one day later with a high dose of 400µg OVA protein (Profos, endotoxin-free). Animals were injected a second time with OVA using the same dose and route after 3 days (short interval) or 11 days (long interval). **(A)** Five days after the last OVA injection FACS analysis was performed to determine the percentage of DO11.10 T cell recovery by KJ1-26 staining among CD4^+^ living spleen cells, gated as in Figure 5b but without CFSE. **(B–D)** Representative FACS analysis by staining CD4, KJ1-26, iCTLA-4 and Foxp3 and statistical evaluation of Treg subsets. **(E–G)** CD4^+^ splenic T cells of each mouse group were magnetically separated and tested for their suppressive capacity by culturing them with proliferating CFSE-labelled responder CD4^+^ T cells. **(E)** Example of CFSE dilution from each animal group. Numbers represent the number of cell divisions. **(F, G)** Summary of e showing all groups separated into early cell divisions (1 + 2) and later divisions (3 – 5), respectively. (Error bars: SD, Dots represent individual mice. One-way ANOVA with multiple comparisons *p < 0.05, **p < 0.01, ***p < 0.005, ****p < 0.0001). ns, not significant.

Next, we analysed whether the DO11.10 T cells expanded, developed into Foxp3^+^ or Foxp3^-^ cells and showed regulatory properties. While the short interval led to an increased frequency of splenic DO11.10 T cells, the long-term interval frequency remained almost at the level of control mice (no OVA) (**Figure 6A**). Additional staining for iCTLA-4 and Foxp3 indicated that the frequencies of CD4^+^ KJ1.26^+^ Foxp3^+^ Treg (**Figures 6B, C**) as well as CD4^+^ KJ1.26^+^ iCTLA-4^+^ Foxp3^−^ cells (**Figures 6B, D**) were enhanced as compared to not OVA injected controls. However, as predicted, the short-term protocol clearly favoured the generation of CD4^+^ KJ1.26^+^ iCTLA-4^+^ Foxp3^−^ cells over CD4^+^ KJ1.26^+^ Foxp3^+^ Treg, while CD4^+^ KJ1.26^+^ Foxp3^+^ Treg expansion was more prominent with the long term interval (**Figures 6C, D**).

We further tested the suppressive capacity of splenic T cells from such treated mice *in vitro*. Since the absolute numbers of remaining DO11.10 cells from a single spleen were too low to isolate them for suppressor assays, we enriched the whole splenic CD4^+^ T cell population for these experiments. The results indicate that only the splenic CD4^+^ T cells of short-term interval OVA-treated mice acquired significant suppressive activity as compared to control and long-term interval OVA-treated mice (**Figure 6E**). Of note, although the long-term protocol induced higher frequencies of Foxp3^+^ Treg cells (**Figure 6C**), this did not result in a detectable suppressive effect (**Figures 6F, G**). In detail, the suppressive effect was more prominent at later cell divisions (3-4^th^ generation) as compared with earlier ones (**Figures 6F, G**). Thus, these results suggest that only short-term interval injections of OVA promote the generation of CD4^+^ KJ1.26^+^ iCTLA-4^+^ Foxp3^-^ cells that are likely to account for the detectable suppressor function in our experimental settings.

## Discussion

Anergy can be induced *in vivo* by a single intravenous injection of high dose antigen and repetitive administration of antigen can induce IL-10 production in these cells (14–16, 19). However, the acquisition of suppressor functions after conversion of anergic non-suppressive T cells into Foxp3^-^ IL-10^+^ suppressive T cells, so-called Tr1 cells, *in vivo* are still not fully understood. Furthermore, markers of anergic T cells and the converted Tr1 cells have not been studied in detail. We therefore sought to characterize the cells at different stages from anergic to IL-10^+^ Tr1 cells and their regulatory function. Together, our data show the induction of several surface markers associated with anergy on naive T cells after a single high dose OVA injection and their further up-regulation during conversion to Tr1 cells after a second OVA injection. At the same time Tr1 cells up-regulate IL-10 as compared with anergic T cells. The functional anergic state as measured by proliferation is maintained in Tr1 cells independent of the time interval between two OVA injection protocols, but suppressor function required a short-term interval where CTLA-4 is still expressed by the cells.

After a single injection of high dose OVA, T cell anergy was induced, as observed by a moderate and very transient expansion of adoptively transferred ITIB.OT-II cells. After a first wave of proliferation, a rapid deletion of antigen-specific cells has been reported in experiments comparing tolerogenic versus immunogenic stimuli *in vivo* (38). High dose antigen injection in form of either OVA peptide or full OVA protein further led to upregulation of several markers associated with T cell anergy. A combination of the surface markers CD28 and PD-1 has been proposed to identify anergic T cells and to delineate them from naïve or activated T cells (28). In our experiments CD28 and PD-1 expression were slightly upregulated upon high dose antigen injection. Furthermore, a naturally occurring population of anergic CD4^+^ T cells has been described as Foxp3^−^ CD44^high^ CD73^high^ FR4^high^ (20). In our model, Foxp3 expression was not induced in adoptively transferred OT-II cells whereas CD44, CD73 and FR4 were all upregulated upon only one high dose OVA injection. Upregulation of the memory marker CD44 in response to antigen encounter is expected whereas CD73 and FR4 have been described for Foxp3^+^ Tregs (39, 40). Although FR4 has been reported to be essential for natural Tregs maintenance, its precise function remains unclear (40). CD73 acts together with CD39 and plays an important role in regulating extracellular ATP, which is released at sites of tissue damage and inflammation (41). Adenosine, which is finally generated in this process has been shown to exhibit numerous immunoregulatory activities (42). CD73 has therefore been proposed as an additional pathway by which both classical CD25^+^ Foxp3^+^ Tregs but also CD25^−^ T cells can perform suppressive and anti-inflammatory functions (39). In our case, injected OT-II cells upregulated intracellular CTLA-4 and the transcription factor Egr-2, both of which have been shown to play important role in anergy induction and maintenance (24, 29, 43).

Several analyses revealed a dominant role for the early growth response genes 2 (Egr-2) and 3 (Egr-3) for expression of the anergy-associated transcriptional program, as identified in clonal anergy and in *in vivo* anergy models (8, 28, 29). The functional requirement for Egr-2 expression for T cell anergy has been demonstrated also *in vivo* (44) and Egr-2 induction was dependent on TCR-mediated NFAT signalling (45). Therefore, Egr-2 serves as one of the best markers for anergic T cells.

CTLA-4 is required for the conversion of anergic T cells into Tr1 cells *in vitro* and Egr-2 is crucial for IL-27-dependent differentiation into IL-10^+^ Tr1 cells (23, 46, 47) indicating that both factors prime anergic cells for conversion into Tr1 cells by a single antigen injection. The functional role for Tr1 conversion or suppression of all these different markers was not further investigated here and remains to be determined by future studies.

Alike CD28, CD25, which was upregulated shortly after the first antigen encounter, was not significantly induced at any time point after the second stimulus. This marks a clear-cut difference to the *in vitro* findings using immature DC for Tr1 conversion when the converted cells expressed high levels of CD25 and employed IL-2 consumption as a suppressor mechanism (23). However, impaired re-expression of CD25 in *in vivo* anergized T cells has been described previously (48). Since characteristics and mechanisms of anergy induction (especially regarding the role of CTLA-4) and anergy reversal largely differ between *in vitro* and *in vivo* models, differences in the conversion or reactivation of anergic T cells are also likely.

Besides surface marker expression, in our DO11.10 system, the cells showed reduced proliferative capacity, the hallmark of T cell anergy (3). In the OT-II system, functional anergy could not be tested due to their disappearance by unspecific deletion but may be suggested by their phenotypic characteristics and the lack of IL-10 production after the first injection (4). Our *in vitro* data indicated that a second antigenic stimulus has to occur within a short time span in which high CD28 and CTLA-4 expression is a prerequisite to foster the conversion of anergic T cells to Tr1 cells with suppressive capacity (23). Interestingly, the marker phenotype of Tr1 cells induced from both OT-II and DO11.10 cells remained quite similar after the second OVA injection, pointing out the anergic phenotype. This, however, does not conflict with the ‘regulatory effector Tr1 phenotype’ described here since IL-10^+^ Foxp3^−^ Tr1 cells and even Foxp3^+^ Tregs have been shown to be anergic, probably to prevent the premature inhibition and shut-down of the early immune response (4).

Anergic T cells expressing both CD73 and FR4 have been shown to convert into Foxp3^+^ Tregs *in vivo* (20). In our OT-II experiments, however, CD44^+^ CD73^+^ FR4^+^ cells generated by a single high dose OVA injection rather converted into Foxp3^−^ IL-10^+^ Tr1 cells upon a second antigen injection within a short-time window while the frequency of classical Foxp3^+^ Tregs remained low. Foxp3^+^ Tregs can be induced *in vivo* by continuous application of low dose antigen by osmotic minipumps and TGF-β from steady-state migratory DC (49). In contrast, high dose antigen application as used in our anergy model may exceed the capacity of the endogenous TGF-β in the antigen presenting system to provide enough of this cytokine for Treg conversion, thereby blocking Treg induction and instead promoting the generation of Tr1 cells. In the DO11.10 system, we also observed an increase in Foxp3^+^ cells using the short-term protocol. This may be a characteristic of the BALB/c mouse strain, which has been shown to generate higher proportions of Foxp3^+^ cells than C57BL/6 mice also in other settings such as influenza virus vaccination (50).

The key players for Tr1 conversion in the *in vitro* setting using immature DCs were CTLA-4 and CD28 (23). We could show that injection of high dose antigen leads to an upregulation of iCTLA-4 in both adoptively transferred OT-II and DO11.10 cells and that a second stimulus shortly after the first one allowed for conversion into Tr1-like cells in the OT-II setting. To test whether this conversion critically depends on CTLA-4, we aimed to perform the second OVA injection at time points when anergic cells exhibited low CTLA-4 expression. Unfortunately, this was not possible in the OT-II system since anergic T cells were rapidly deleted in this setting. In contrast, anergic DO11.10 cells seemed to be more stable *in vivo* allowing a longer follow-up time period. Tr1 conversion in the OT-II system was mainly determined by the secretion of IL-10. However, since the IL-10 reporter mouse ITIB was only available on C57BL/6 background, we were not able to confirm these findings in the DO11.10 system. Nonetheless, we confirmed regulatory function using suppression assays and found that only cells generated with the short-term injection protocol displayed a suppressive capacity supporting our hypothesis that CTLA-4 is crucial for this process. Of course, dependency on CTLA-4 and potentially also CD28 as well as other mechanisms such as ATP degradation and the requirement of Egr-2 need to be confirmed by further studies. These, however, were beyond the possibilities of our system here, that did not allow clearly to address the molecular mechanisms underlying our observations.

Although anergy induction has been classically performed with CD3 simulation without costimulation, this may not reflect the *in vivo* situation of antigen-presenting cells. In contrast to antigen-experienced T cell clones, naïve CD4^+^ T cells were resistant to anergy induction *in vivo* and *in vitro* by anti-CD3/TCR ligation in the absence of costimulation (51). This was not observed when we used immature bone marrow-derived DC for anergy induction (52). The two facts that anergy-inducing MHC II^low^ immature BM-DCs express low levels of CD80 but not CD86 (53) and that CD80 shows a higher binding affinity to CTLA-4 than CD28 (54) may indicate a role for a DC/CD80 to T cell/CTLA-4 interaction. In fact, anergy induction of naïve CD4^+^ T cells appeared to be additionally dependent on B7 costimulation-driven CTLA-4 engagement (55). The precise role and signalling mechanisms of CTLA-4 for anergy induction in naïve T cells is still a matter of debate (56). Earlier reports suggested that CTLA-4 signalling prevents cell cycle progression through regulation of the cyclin-dependent kinase (cdk) inhibitors p27^Kip1^ and p21^Cip1^ (24, 57), although we found that this may not be a strict requirement (58). Here, we found a clear correlation between the acquisition of suppressor function of Tr1 cells and their up-regulation of CTLA-4. Whether CTLA-4 is required for conversion of anergic into Tr1 cells as observed before *in vitro* (23), or employed as a suppressor mechanism by controlling CD28 signaling (59) requires further investigations.

An important suppression mechanism of both Foxp3^+^ Tregs and Tr1 cells generated from anergic T cells *in vitro* is the consumption of IL-2 by the expression of a high affinity IL-2 receptor (23, 60). The same mechanism applied for peptide-induced tolerant cells when they were tested in suppression assays *in vitro*. However, suppressive capacity of the same cells *in vivo* depended on IL-10 production (61). These cells were predominantly CD25^−^ and CTLA-4^+^ thereby showing a similar Tr1 phenotype generated in our models. IL-10 deficiency has furthermore been shown to play an important role for an IL-10-dependent feedback-control and host protection during infection (62–64). This implies an important role for IL-10-dependent suppression mediated by Tr1 cells especially *in vivo*. In this respect, is has been reported that a rapid *in vivo* expansion of IL-10-producing Tr1 cells by repetitive natural high dose bee venom exposure of beekeepers during beekeeper season downregulated local skin allergic responses and eventually conferred allergen tolerance (65). Therefore, it is tempting to speculate that high dose allergen challenge in short time intervals – as demonstrated here - might be the mechanistic basis to provide a fast and effective way to deliver naturally occurring T cell tolerance to allergens by Tr1 cells in nonallergic healthy humans.

Here we characterized a population of anergic CD4^+^ T cells that develops from naïve T cells after antigen encounter without costimulation. These cells expressed CD44, CD73, FR4, iCTLA-4 and Egr-2 and can be reactivated by a second antigenic stimulation. Upon this second stimulus within a short time interval, the cells maintained their anergic phenotype, started to secrete IL-10 (a characteristic feature of Foxp3^−^ Tr1 cells) and simultaneously suppressed effector cell proliferation. This may indicate a role for persisting anergic T cells as a memory cell pool for Foxp3^−^ Tr1 cells *in vivo*. The long-term protocol for the OVA injections led to persistence of the anergic state but not the acquisition of regulatory functions. A deeper understanding of the mechanisms that allow for reactivation of this memory compartment will be useful for the development of optimized vaccination protocols that may allow for prevention or suppression of autoimmune or allergic diseases.

## Data Availability Statement

The raw data supporting the conclusions of this article will be made available by the authors, without undue reservation.

## Ethics Statement

The animal study was reviewed and approved by Regierung von Unterfranken, AZ 52/14.

## Author Contributions

AT, TS, LC, and IE performed the experiments and analysed the data. AT, AK, and ML compiled the data and wrote the manuscript. All authors contributed to the article and approved the submitted version.

## Funding

This work was supported by the DFG grant LU851/14-1 for MBL and the Interdisciplinary Center for Clinical Research (IZKF) project A-408 for AK and MBL and AdvCSP-2 for AK. We thank Marion Heuer for her expert technical assistance on the project. This publication was supported by the Open Access Publication Fund of the University of Würzburg.

## Conflict of Interest

The authors declare that the research was conducted in the absence of any commercial or financial relationships that could be construed as a potential conflict of interest.
